# Novel Anisotropic Ductility of a High Strength Annealed Ti-20Zr-6.5Al-4V Alloy

**DOI:** 10.3390/ma11040529

**Published:** 2018-03-30

**Authors:** Bing Zhang, Xing Zhang, Yuanzhi Jia, Xinyu Zhang, Mingzhen Ma, Riping Liu, Qin Jing

**Affiliations:** State key laboratory of Metastable Materials Science and Technology, Yanshan University, Qinhuangdao 066004, China; xingzhang9024@yeah.net (X.Z.); ysujyz@126.com (Y.J.); xyzhang@ysu.edu.cn (X.Z.); mz550509@ysu.edu.cn (M.M.); riping@ysu.edu.cn (R.L.)

**Keywords:** Ti-20Zr-6.5Al-4V alloy, uniaxial tensile, similarly orientated α laths, crack, ductility anisotropy

## Abstract

In this work, we investigate the mechanical properties of an annealed high strength Ti-20Zr-6.5Al-4V alloy in uniaxial tensile tests in different directions. The results show that the alloy exhibits obvious anisotropic ductility in different directions, while the tensile strength of the alloy remains almost unchanged. This phenomenon is closely related to α laths with similar orientations along the prior-β grain boundaries. These α laths significantly affect the initiation and propagation of cracks when the alloy reaches its yield limit, thereby affecting the ductility of the alloy, such that it exhibits anisotropic ductility.

## 1. Introduction

Ti alloys are applied in many fields due to their many excellent properties such as exceptional strength-to-weight ratio, high performance at elevated temperatures, high hardenability, outstanding corrosion resistance, good biocompatibility and excellent fatigue propagation properties [1,2,3,4,5]. However, their poor tribological properties and high thermal expansion coefficient restrict their application [6,7], especially as structural materials in the extreme space environment.

Recently, Zr has been noted for its ability to improve the mechanical properties of Ti alloys and a new Ti alloy comprising Ti-20Zr-6.5Al-4V (wt %, shortened to 20Zr hereafter) has been developed [8]. The alloy exhibits higher tensile strength and hardness than the well-known Ti-6Al-4V alloy [8,9]. Thus, 20Zr alloy is an excellent candidate as a structural material for application in the space environment.

In practice, the anisotropy of mechanical properties must be considered when designing and building structural components. Previously, researchers have conducted a series of studies on the 20Zr alloy [10,11,12,13,14]. However, as yet, no study has focused on the anisotropy of the mechanical properties of 20Zr alloy. As a candidate structural material for use in the space environment, alloys must be heavily tested with respect to their reliability. Thus, it is necessary to investigate the anisotropy of mechanical properties of this alloy.

Most studies of the anisotropy of mechanical properties select the rolled or extruded state as the study object because it typically reveals the obvious anisotropy of mechanical properties [15,16]. However, in this study, we selected 20Zr alloy annealed at 750 °C for 2 h to investigate the anisotropy of the mechanical properties because it exhibits a balance of high strength and ductility [13]. This alloy is in a dual (α + β) phase in this condition. The results from this study may help us to understand the failure process of the 20Zr alloy, and give us some useful information to enhance the mechanical properties of the alloy.

## 2. Materials and Methods 

**Figure 1:**
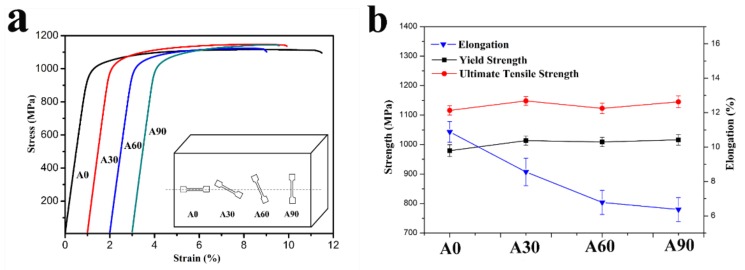
Uniaxial tensile test results of the different-direction samples. (**a**) Engineering stress-strain curves of the samples. Inset illustrates the sample cut method used. (**b**) Elongation, yield strength, and ultimate tensile strength of the samples.

The preparation process of the 20Zr alloy used in this study has been presented in elsewhere [13]. The alloy sheet used in the experiment was cut lengthwise from a long round bar. The round bar has been processed by forging prior to the annealing treatment. We cut the tensile test samples from the same alloy sheet with certain angles between the center axis of the samples and the alloy sheet, as shown in the inset of Figure 1a. According to these angles, we labeled the samples as A0, A30, A60 and A90. These specimens were dog-bone shaped with a gauge size of 2 × 3× 15 mm^3^. We performed all the tensile tests on an Instron 5982 mechanical test system with a crosshead speed of 0.375 mm min^−1^. We repeated the tensile test for each angle three times to reduce random error. We observed the fracture surfaces of these samples using a Hitachi S3400N scanning electron microscope (SEM, Hitachi, Tokyo, Japan). To identify the microstructure details of the samples, we examined the cross sections perpendicular to the tensile direction using an EDAX-TSL electron backscatter diffraction (EBSD) system (Ametek, Berwyn, PA, USA). In addition, to identify the propagation paths of the cracks in the tensile testing samples, we cut a fractured sample longitudinally along the tensile direction and examined it by EBSD. Using a standard metallographic procedure, we mechanically polished all the EBSD samples to a 0.5-μm diamond paste, then further improved the samples by vibration polishing. Lastly, we realized the required surface finish by performing electro-polishing in a solution of 10% perchloric acid, 20% butyl cellosolve, and 70% methanol at 20 V and −20 °C. We performed EBSD mapping at an operating voltage of 20 kV at a working distance of approximately 15 mm. We then analyzed the EBSD data using TSL OIM Analysis 7 software.

## 3. Results and Discussion

Figure 1a presents the tensile curves of the samples with different angles, which shows only small variations in the ultimate tensile and yield strengths of the samples. The tensile and yield strengths are about 1100 MPa and 1000 MPa, respectively. Therefore, we can consider the strength of the samples to be unchanged. However, we note that the elongation of the samples varies over a wide range (Figure 1b). This elongation shows a decreasing trend as the angles increase. The elongation of the samples decreased from 10.8% in A0 to 6.1% in A90. This 43.5% reduction in elongation (ductility) indicates that the annealed 20Zr alloy exhibits anisotropic ductility.

**Figure 2:**
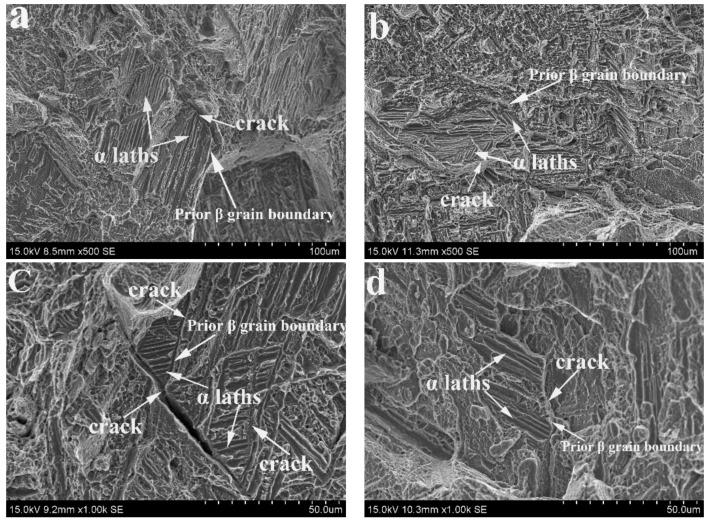
Typical morphologies of the cracks on the fracture surface of the different-direction samples after tensile testing: (**a**) A0; (**b**) A30; (**c**) A60; and (**d**) A90.

To identify the mechanism of the fracture, we used SEM to observe the fracture surface of the different-direction samples. Figure 2 shows the typical morphologies of cracks that appeared in the fracture surface of these samples. We noted that these cracks tend to initiate at and extend along the prior-β grain boundaries. In addition, some α laths with a similar orientation were distributed along the cracks. The process of determining α laths has been described in previous work [13]. The α laths on the fracture surface can be determined by comparing the size and morphology.

**Figure 3:**
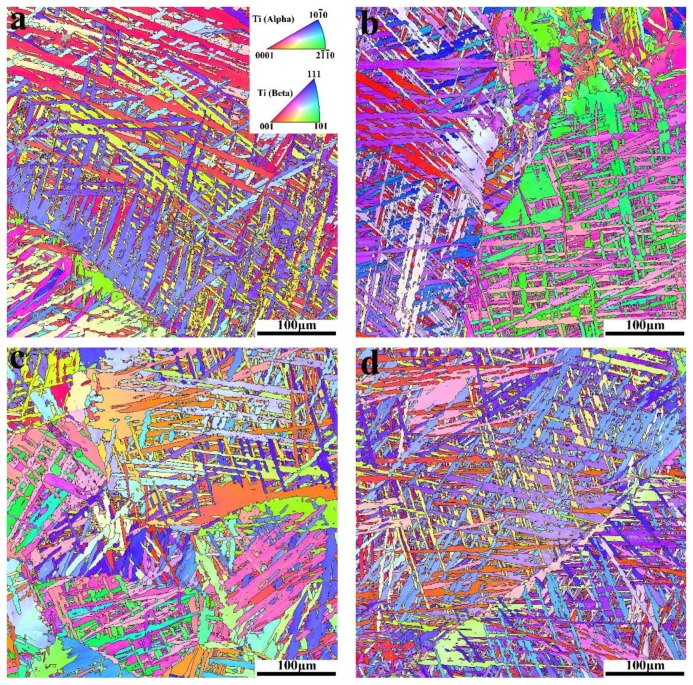
IPF maps of the different-direction samples: (**a**) A0; (**b**) A30; (**c**) A60; and (**d**) A90.

We investigated the microstructure of the samples prior to the tensile test using EBSD, the results of which are shown in Figure 3. The inverse pole figure (IPF) maps show that in both samples, there are some α laths with similar orientations precipitated along the prior-β grain boundaries. In addition, the orientations of these similarly orientated α laths areas are different from each other.

**Figure 4:**
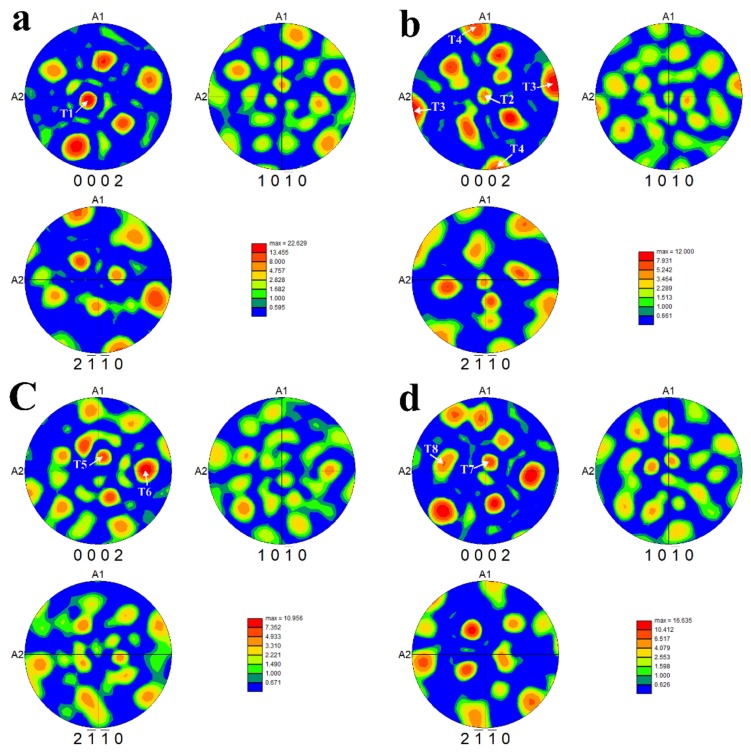
Pole figures of the samples in different-directions: (**a**) A0; (**b**) A30; (**c**) A60; and (**d**) A90.

Material properties have been found to be significantly affected by texture [17]. To reveal the texture of the samples in different directions, we use pole figures to characterize the texture of α phases (87.3%, in volume fraction) in the samples. Figure 4 shows {0002}, {10$\bar{1}$0} and {2$\bar{1}$$\bar{1}$0} pole figures of the samples in different directions as shown in Figure 3, whereby the tensile direction is parallel to the normal direction (ND). It can be seen that the maximum texture intensity shows a tendency to decrease and then increase with increase in the angles between the center axis of the samples and the alloy sheet surface. Specifically, the maximum texture intensity of samples A0, A30, A60, and A90 are 22.629 MRD (multiple random distributions), 12 MRD, 10.956 MRD, and 16.635 MRD, respectively. In {0002} pole figure of sample A0, there are several texture components with high intensities. Besides a strong basal fiber texture (about 21 MRD, marked with T1) of about 15° shift from ND toward A2, the other texture components formed a parabolic shape. In sample A30, a basal fiber texture with a relatively low intensity (about 5 MRD, marked with T2) can be seen, and a strong texture component (about 12 MRD, marked with T3) rotates around ND at about −10° from A2 to A1. Besides these texture components, a component of relatively low intensity (about 7 MRD, marked with T4) rotates about −10° around ND from A1 to A2. In addition, five texture components with relatively low intensities around the basal texture component are formed in the {0002} pole figure. There is a complex texture state in A60, in which many texture components are formed. A relatively weak basal fiber texture (about 7 MRD, marked with T5) shifts about 20° from ND toward A1, and a relatively strong texture component (about 10 MRD, marked with T6) shifts about 65° from ND toward A2 in {0002} figure. In sample A90, a relatively strong basal fiber texture (about 10 MRD, marked with T7) shifts about 15° from ND toward A1, and a relatively weak texture component (about 7 MRD, marked with T8) rotates about 10° around ND from A2 to A1. The other texture components formed a parabolic shape similar to the texture components formed in sample A0. The numerous texture components that appear in the samples of different directions are related to the variants selection during the β→α transition. The similar α laths orientations are attributed to the high intensity of the texture components.

**Figure 5:**
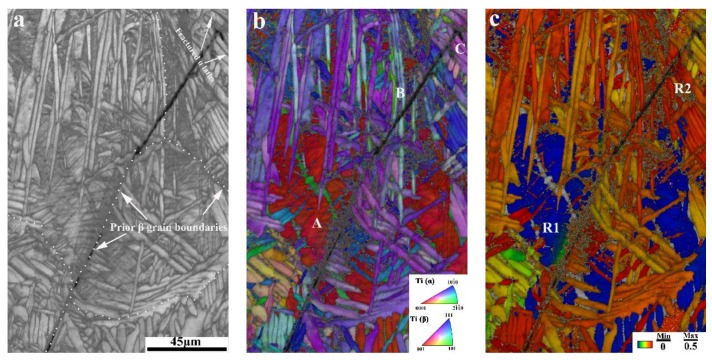
EBSD analysis results of the fractured sample after tensile testing: (**a**) IQ map; (**b**) overlay of IQ and IPF maps; (**c**) overlay of SF distribution and IQ maps.

To identify the crack propagation paths, we also used EBSD to examine the fractured sample after conducting the tensile test. As shown in Figure 5a, in image quality (IQ) map, the crack originated from the prior-β grain boundary and extended through the parallel α lath areas. Previous research shows that α variants inherited from the same parent β grain are characterized by specific misorientations linked to the Burgers orientation relation [18]. Thus, EBSD analysis can be employed to identify the prior-β grain boundaries. Figure 5b shows an overlay of the IPF map and IQ maps, in which we see the parallel α laths areas (marked with A, B, C) have similar orientations (similar colors). These results are consistent with those of the fracture surface analysis. In our previous study of 20Zr alloy, we observed no twins in the annealed sample following tensile test [13]. As such, slip can be considered to be the dominant deformation mode in the tensile test. In HCP (hexagonal close-packed) alloys, there are three slip systems with a <$\bar{2}$110> Burgers vector (a-type), namely, the {0001} basal slip, {10$\bar{1}$0} prismatic slip, and {10$\bar{1}$1} first-order pyramidal slip [19]. Some researchers have shown that a prismatic slip is easier to activate in Ti-6Al-4V alloys at room temperature than the other two slip systems, because prismatic slip has the lowest critically resolved shear stress (CRSS) [19,20,21]. Therefore, in this study, we considered prismatic slip to be the main slip system at room temperature in 20Zr alloy, which has a composition similar to Ti-6Al-4V alloy. Figure 5c shows Schmid factors (SF) distribution map of the area shown in Figure 5a. In this map, we set the slip system as prismatic slip. The SF distribution shows that some similarly orientated α laths alongside the cracks are blue and green in color (marked with R1), which means that they are in “hard” orientation. This indicates that these areas are less prone to plastic deformation under specific stress conditions, so when subjected to tensile stress, these areas most likely release energy through the formation of cracks. In addition, it can be seen from Figure 5c that the cracks continuously extend through some similarly orientated α laths area (marked with R2) in red color (high-SF-value, “soft” orientation). This is because cracks extend through the similarly orientated α laths areas without change direction, thereby consuming the least amount of energy [22]. This explains why the cracks initiate at the prior-β grain boundaries and extend through the similarly orientated α laths areas.

**Figure 6:**
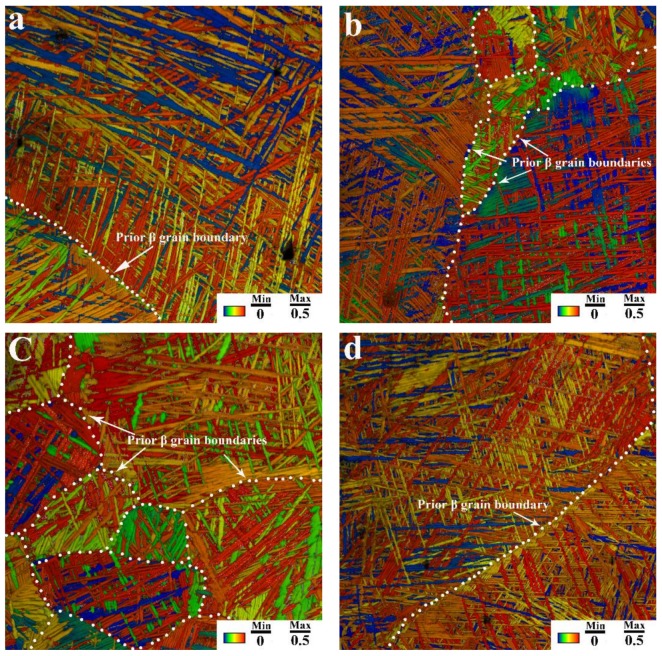
Overlay of SF distribution and IQ maps of the different-direction samples shown in Figure 3: (**a**) A0; (**b**) A30; (**c**) A60; and (**d**) A90.

Figure 6 shows an overlay of the IQ and SF distribution maps of the different-direction samples shown in Figure 3, in which we also set the slip system as prismatic slip. This map shows that in different-directions samples, the numbers and area fractions of the low-SF-value and similarly orientated α lath areas distributed alongside the prior-β grain boundaries (indicated by white dot lines) are quite different. This is due to selection of α variants in different directions. As shown in Figure 5, these similarly orientated α lath areas are potential crack initiation and propagation areas, which significantly affect the crack initiation and propagation behavior of the alloy during tensile testing. In sample A0, the cracks are difficult to initiate due to the “soft” orientations (high-SF-value) of the parallel α laths along the prior-β grain boundary. When the parallel α laths that distributed on both sides of the prior-β grain boundaries are in “hard” orientations (low-SF-value), the cracks are more prone to initiate and spread rapidly. The number of the cracks initiated in sample A30 is less than that in sample A60. And in sample A90, due to the “hard” orientation (low-SF-value) of α laths in both sides of the prior-β grain boundary, a long crack along the prior-β grain boundary will appear and spread rapidly. Thus, when the tensile stress reaches the yield limit, the number of cracks initiated and the rate of crack propagation vary due to the inconsistent orientations of the α laths along the prior-β grain boundaries, which in turn cause the different ductility in different directions, while the tensile strength remains unchanged.

In summary, α laths with similar orientations that are distributed along the prior-β grain boundaries significantly affect the crack initiation and propagation of the annealed 20Zr alloy during uniaxial tensile testing, and then affect the ductility of the alloy in different directions, thereby macroscopically exhibiting anisotropic ductility.
